# Breaking Snake Camouflage: Humans Detect Snakes More Accurately than Other Animals under Less Discernible Visual Conditions

**DOI:** 10.1371/journal.pone.0164342

**Published:** 2016-10-26

**Authors:** Nobuyuki Kawai, Hongshen He

**Affiliations:** Department of Cognitive Science, Graduate School of Information Science, Nagoya University, Chikusa-ku, Furo-cho, Nagoya, JAPAN, 464-8601; University of Toyama, JAPAN

## Abstract

Humans and non-human primates are extremely sensitive to snakes as exemplified by their ability to detect pictures of snakes more quickly than those of other animals. These findings are consistent with the Snake Detection Theory, which hypothesizes that as predators, snakes were a major source of evolutionary selection that favored expansion of the visual system of primates for rapid snake detection. Many snakes use camouflage to conceal themselves from both prey and their own predators, making it very challenging to detect them. If snakes have acted as a selective pressure on primate visual systems, they should be more easily detected than other animals under difficult visual conditions. Here we tested whether humans discerned images of snakes more accurately than those of non-threatening animals (e.g., birds, cats, or fish) under conditions of less perceptual information by presenting a series of degraded images with the Random Image Structure Evolution technique (interpolation of random noise). We find that participants recognize mosaic images of snakes, which were regarded as functionally equivalent to camouflage, more accurately than those of other animals under dissolved conditions. The present study supports the Snake Detection Theory by showing that humans have a visual system that accurately recognizes snakes under less discernible visual conditions.

## Introduction

Effective detection of potential threats is crucial for survival. Humans are extremely sensitive to evolutionarily fear-relevant animals (i.e., snakes) [1, 2]. Humans form associations between pictures of snakes and electric shocks more strongly than between pictures of guns and shocks, despite the fact that guns and knives are more dangerous than snakes in the modern environment [3]. Humans find pictures of evolutionarily fear-relevant stimuli (snakes or spiders) more quickly than those of neutral stimuli (flowers or mushrooms) in visual search tasks [4, 5]. Young children with relatively little prior exposure to snakes or their representations also react faster when identifying snakes than other animals [6], which suggests that prior experience with snakes may not play a major role in enhanced human sensitivity [7, 8, 9].

Evidence for an evolutionary specialized attention to snakes comes from our studies with macaque monkeys [10, 11]. Kawai and Koda [11] compared reaction times for snake-naïve macaque monkeys (*Macaca fuscata*) to detect deviant pictures of snakes and spiders in the background of non-threatening animal pictures (koala) and found that quicker detection occurred for snake pictures but not for spider pictures. Despite the fact that monkeys in these studies [10, 11] were reared in captivity and assumed not to have been exposed to real or toy snakes, they reacted to snake pictures quickly. These results with visual search tasks suggest that evolution equipped our primate ancestors with a visual system predisposed to respond to snakes [1, 2, 7, 10, 11, 12, 13], as Darwin pointed out [14].

These results are consistent with the Snake Detection Theory (SDT), which proposes that the need to detect dangerous snakes provided strong evolutionary pressure that resulted in the origin of primates via expansion of their visual system [2, 15]. The first snakes to prey upon primates appeared about 100 million years ago and were constrictors. About 60 million years ago, venomous snakes arose [16, 17], and this favoured further expansion of anthropoid primate visual systems, such that today, anthropoid primates, including humans, are expected to be better able to detect snakes than are other mammals, particularly snakes that are immobile and camouflaged [15]. It might be expected that humans can recognize camouflaged snakes more accurately than other camouflaged animals all else being equal. A series of recent studies has shown that humans are more capable of detecting snakes compared to other stimuli in cluttered or distracting conditions, which simulate the visually demanding conditions in which camouflage occurs [18–21]. It is, however, still unknown whether humans can effectively detect less discernible snakes from a similar background. Here, we examined whether humans are more capable of detecting snakes compared to cats, birds, and fish under highly demanding visual conditions that simulate the ability of snakes to camouflage themselves.

**Fig 1:**
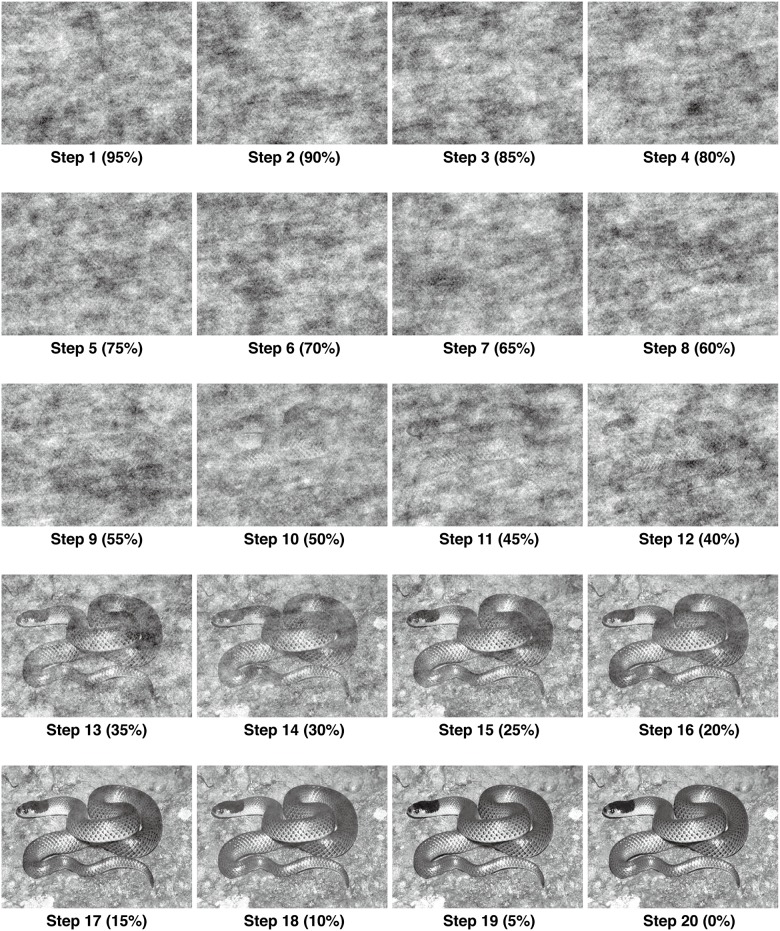
Examples of a Random Image Structure Evolution (RISE) sequence for snake pictures. Includes a sequence of 20 pictures with interpolation ratio starting from 95% to 0% with steps of 5%. RISE sequence will gradually change from unorganized to well discernible.

In this study, we interpolated animal pictures with random noise in various degrees producing different levels of "camouflaged" images, and investigated whether snake pictures were detected more effectively compared to other non-threat animals (birds, cats, and fish) under fragmented perceptual conditions. We used the interpolated pictures (Fig 1), instead of real camouflaged snake images, because of the difficulties of matching the degree of camouflage across natural images of camouflaging animals.

To produce "camouflaged" images, a technique called Random Image Structure Evolution (RISE) was adopted [22]. This algorithm probes multiple responses to dense units of image stimuli by controlling a lot of important low-level visual information. The first step to produce a dissolved image by a RISE technique is to perform an analysis of Fourier transformation analysis for the original image. For any signals (waves) in time space, the Fourier transformation decomposes it from time into the frequencies space. The Fourier transformation coefficient is usually represented as having two elements: magnitude spectrum and phase spectrum. Altering phase information can change the spatial structure, so the second step is achieve a randomized phase spectrum progressively while keeping the original intact magnitude spectrum the same. Finally, an inverse Fourier transformation needs to be manipulated, after combing the randomized phase spectrum and original intact magnitude, the RISE stimuli is produced. A RISE technique produces a continuous sequence, which evolves from a random noisy start and then gradually changes into increasingly more discernible patterns (Fig 1). According to Sadr and Sinha (2004), RISE actually can be thought as a special type of morphing technique, or degradation procedure [22]. The key point is to change phase information which can be controlled parametrically while keeping the low-level visual image properties of the original image, such as spectrum, color distributions, spatial frequency amplitude, and overall luminance, perfectly preserved. As the low-level visual information holds the original properties of an image, a RISE technique takes a great advantage of object recognition without contamination of changes in low-level visual information.

If humans' visual system is preferentially sensitive to snakes, the dissolved images of snakes will be discerned more effectively even under conditions of less perceptual information. Note that this procedure can avoid the major criticism of the procedural problems of the visual search task: attentional bias toward snakes is achieved by engagement with the snake targets or by slow disengagement from distractor snakes. This task simply required detection and identification of the target without any need either to disengage attention from distractors or to move it to the spatial location of the target.

## Material and methods

### Participants

Twenty undergraduates (13 men and 7 women) participated in this experiment. Their age ranged from 18 to 26 years (mean = 20.6, *SD* = 2.72). They were recruited from our subject pool system. All participants were shown printed images of the stimuli used in this study upon recruitment and reported that they were not fearful of these types of animals. They had normal or corrected to normal vision and were right-handed according to their self-report. Informed consent was obtained from all participants.

### Ethics Statement

All participants were paid for their participation and provided written informed consent in accordance with procedures. The methods were carried out in accordance with to the principles expressed in the Declaration of Helsinki and with relevant guidelines and regulations. The study was approved by the Ethics Committee of the Graduate School of Information Science at Nagoya University. Written informed consent was obtained before participation.

### Stimuli

Four animal categories including snake, bird, cat, and fish pictures of high quality on a natural background were converted into grayscale pictures with luminance approximately equivalent for all (see S1 File). Each category consisted of four different pictures. All the animals of the original pictures were clearly depicted. The picture size was 400 × 300 pixels, and stimuli were presented on a 24-inch LCD monitor at a distance of about 60 cm from the participant (16.5 × 12.1° in a visual angle).

The series of images were produced from the original pictures by using the random image structure evolution (RISE) paradigm [18]. RISE sequence parametrically randomized phase spectrum only to alter the spatial structure of the original images by which the original magnitude spectrum and luminance and contrast were kept constant. Each RISE sequence ranged from 95% to 0% interpolation ratio of the original phase spectrum in steps of 5%, resulting in 20 images for each RISE sequence. The interpolation ratio of 100% represents a complete random phase spectrum, while the interpolation ratio of 0% represents a no-change (original) phase spectrum (Fig 1). This technique resulted in a continuous changing pattern from unorganized to well discernible. Note that RISE has an important advantage of keeping the low-level perceptual information of the original stimuli; all the images that belong to a sequence have the same magnitude spectrum and overall luminance and contrast [20].

We adopted the RISE paradigm because previous studies showed that physically abused children, who were presumed to have experienced high levels of threat and hostility, accurately identified facial displays of anger on the basis of less sensory input than did typically developing children in the RISE paradigm [23]. We predicted that if the participants are sensitive to specific stimuli (i.e., snakes), then they will discern those stimuli more accurately than other stimuli (Fig 1, S2 File).

### Procedure

The participants were required to fix their chin on a chin rest in a sound attenuated room with dim light, and put their hands naturally above a response box (Cedrus, RB-730) to press the buttons in accordance with the arranged positions of animal names, where the names were labeled correspondingly. The stimuli were presented in the center of the screen with a black background for 3 sec. As soon as pictures were invisible by a flash of masking screen, the participants were required to press one of four buttons during the following 3-sec black screen period as correctly as possible irrespective of their confidence level.

Before the test, the participants were accustomed to this procedure with other RISE sequences of four categories of animals (horse, dog, elephant, and koala) until they mastered this procedure. A RISE sequence was presented continuously from unorganized to well discernible resulting in 20 steps in one sequence. In the test, a total of 16 blocks were presented randomly (four snake, four bird, four cat, and four fish blocks). For each stimulus block, a RISE sequence, which contained 20 pictures ranging from 95% to 0% interpolation of original pictures, was presented continuously resulting in 20 trials in one stimulus block. Each participant received 16 blocks and 20 trials per block (320 trials in total).

## Results

**Fig 2:**
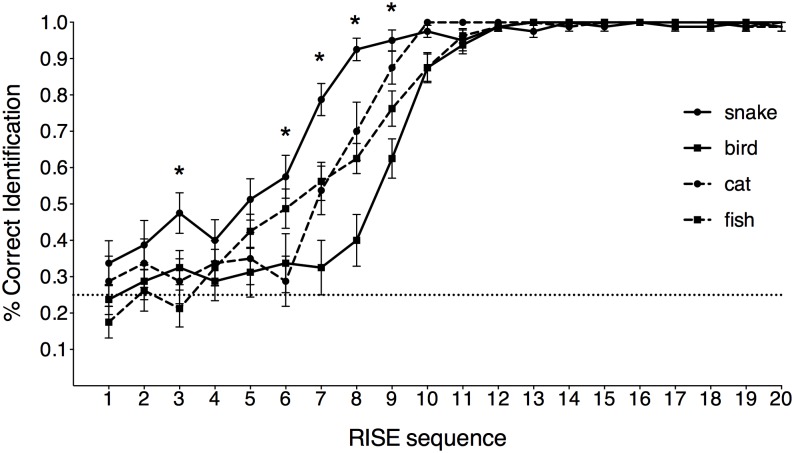
Mean identification rates for each type of stimuli (snake, fish, cat, bird). Asterisks on the line graph indicate significantly different accuracies from ANOVA results. Chance performance (0.25) is indicated by the horizontal dotted line. Error bars denote *SEM*.

Fig 2 illustrates the mean recognition accuracy for four animal categories. A two-way analysis of variance (ANOVA) of 4 stimuli (snake, fish, cat, bird) × 20 steps (RISE sequence) was applied to the accuracy data. Although all the recognition accuracy levels for these animals increased as the RISE sequence progressed, these accuracies were varied at highly dissolved images (*F*(19, 361) = 359.118, partial *η*^2^ = .950, *p* < .001). As expected, the snake pictures were recognized more accurately at unorganized steps and exceeded 90% correct at step 8 (interpolated 60% by random noise image), while the others were not recognized until at step 10 or later. The overall ANOVA yielded a significant main effect of stimuli (*F*(3, 57) = 4.164, partial *η*^2^ = .180, *p* = .01), and a significant stimuli × step interaction (*F*(57, 1083) = 4.163, partial *η*^2^ = .180, *p* < .001). Post-hoc analyses with Ryan correction revealed that the recognition accuracies of snake images were significantly higher than those of birds at Steps 6 through 9, those of cats at Steps 6 through 8, and those of fish at Steps 7 through 9. The accuracies of bird images were significantly lower than those of fish at Steps 6 through 9 and lower than those of cats at Steps 7 through 9.

## Discussion

The present study demonstrates that snakes were recognized more accurately under less discernible conditions than other animals. We assume that the images interpolated by random noise are functionally equivalent to camouflage in the wild, because the animals of these images are hard to discern from the background. These results are consistent with the Snake Detection Theory’s main argument that primates’ need to visually detect snakes provided a strong selective pressure that ultimately resulted in expanded visual systems [2, 15]. The present study suggests that one way this is expressed behaviorally is in the ability to detect snakes under visually taxing conditions. Human participants in our study were able to discern snake images more accurately in more highly fragmented visual scenes than they discerned images of cats, birds, and fishes. These results are consistent with previous studies reporting that humans are more capable of detecting snakes than other stimuli in cluttered or distracting conditions, which simulate the more visually demanding conditions under which camouflage occurs [21]. Recognition of snakes may be enhanced in environments that are visually heterogeneous, complex, or cluttered with multiple potential distractors (i.e. conditions under which snakes are often found in nature) [24].

Although many studies with human adults [4, 5], children [6, 8, 9], and macaque monkeys [10, 11] from our laboratory and others support the Snake Detection Theory by demonstrating that humans and primates have an attentional bias toward snakes, there are still criticisms that quick detection of snakes in the visual search tasks is ambiguous, specifically, that it may be explained by speeded detection of threat targets (engagement), by slowed disengagement from threat distractors, or both [25]. More importantly, using visual search often generates the possibility of low-level perceptual confounds [26]. Thus, detection of predators by primates seems to be driven by a simple feature, such as dark spots against a brighter background in the case of leopards [27] and many animal species display their teeth as an enraged facial expression, which could bias toward quicker detection of conspecifics’ angry faces [28].

As visual search paradigms are criticized for excessive low-level visual differences between target and distractors [25], New et al. (2007) investigated the evolutionary driven attentional priorities in change detection paradigm under the phenomenon of “change blindness”, in which the participants were exposed to alternations between complex natural scenes and duplicates with a single change which only added an object that was either animal (human, animal) or non-animal (plants, artifacts, including vehicles) [29]. The participants were “change blind” to 34% of the added non-animal objects, whereas they only missed 11% of added animals or humans. They argue that evolutionary adaptiveness of ancestral humans required keeping an eye out for animals and humans in the environment for survival; humans could be potential mates, friends, or foes, and animals could be a meal or a potential threat, while many non-animal objects were also important to humans in the wild (e.g. edible plants), and many animals were innocuous and could be overlooked by primates. These results provided evidence that an attentional priority for detecting the presence of animals is more important evolutionarily for primates. However, in terms of threat perception, both the visual search paradigm and change direction paradigm use unobstructed viewing conditions, but in nature often only partial information exists. Animals that can take advantage of partial information to respond appropriately to predators will have greater chances of survival [6]. Furthermore, these studies are subject to criticisms that they are based on unsystematic sampling of image spaces, which have to ensure that the high-level information, such as meaningful recognition of some visual aspects, are not contaminated by low-level information [22].

We have avoided the pitfalls of previous studies by controlling low-level visual information by parametrically randomizing the phase spectrum based on a RISE technique [22], which produces a continuous sequence that dissolves into an unstructured random field and then gradually forms an organized discernable pattern. The effective detection of snakes in this study cannot be attributed to low-level perceptual features. If any simple features (e.g., specific postures [8], color [9], or elongated body shapes) are critical to quick detection of snakes in the visual search task, the features would not be able to keep their properties in this study, due to the interpolation process. As mean luminance and mean contrast were approximately equalized across images, these variables, including colors and low-level perceptual features, would not contribute to producing the present results. However, as mentioned earlier, the human visual system may also include a high-level, category-specialized, system that monitors “specific” animals by their unique colors, postures, and body shapes. For instance, both human adults and children demonstrate “superior” detection when typical striking posture is displayed by the snake [8]. They can detect coiled objects, including snakes, faster than they can detect flowers, but they cannot detect non-coiled snakes faster than they can detect flowers [30]. Rhesus monkeys use posture to assess the level of risk from snakes [31]. However, coiling was not necessary to elicit a strong reaction. These monkeys responded vigorously to an only partially exposed snake model. In addition, wild vervet monkeys detected only a small portion (less than 2.7 cm) of the unnatural shape (i.e., flat) of the snakeskin and reacted as if they had found a real snake [24]. Snakes are often partially obscured by vegetation and rocks. The salient cue of a snake should be a small unit, such as snake scales, which would contribute to the effective recognition of snakes [24, 31]. Because scales are universal to snakes but otherwise rare in nature [15, 32], they should be a highly reliable visual cue for snake detection. In fact, neurons in the pulvinar nuclei actively respond to diamond-shaped or checkerboard stimuli [33, 34], which are analogous patterns to snake scales [15].

The present study focused on the recognition of snakes, while the fear module hypothesis suggests that both snakes and spiders may be prototypical evolutionarily threat-relevant stimuli [1]. Although human adults have been shown to quickly detect deviant spider pictures among an array of mushroom pictures, this attention bias disappeared when the deviant spider pictures were embedded among animal pictures [35]. Studies with visual search tasks have revealed a larger threat-detection advantage for snakes than for spiders [5, 18]. Our study with three Japanese monkeys show that the monkeys detect a single snake picture among eight non-threat animal pictures (koala) more quickly than vice versa, however no such difference in detection was observed for spiders and non-threat animals [11]. Electroencephalogram studies using early posterior negativity (EPN), which reflects the early selective visual processing of emotionally significant information, also suggest that the degree of EPN for spider pictures was smaller than that for snake pictures and not different from other non-fear-relevant animals [12]. Therefore, attentional bias toward spiders would be weaker than toward snakes. In fact, our pilot study with the RISE paradigm showed that the recognition accuracy for spiders was not higher than those for other insects.

Finally, the poor recognition of birds in this study deserves some mention. Familiarity with birds, cats, and fish would not account for these results, since they are equally familiar to participants. Birds have a distinctive body shape just as cats and fish do. We do not have a plausible explanation for participants’ inaccurate recognition of bird images. Future studies should explore why images of birds are less accurately recognized than those of cats or fish under visually demanding conditions.

## Supporting Information

S1 File The luminance and contrast of each image. (DOC)

S2 File Examples of a RISE sequence for cat pictures. Examples of a Random Image Structure Evolution (RISE) sequence for cat pictures. Includes a sequence of 20 pictures with interpolation ratio starting from 95% to 0% with steps of 5%. RISE sequence will gradually change from unorganized to well discernible.
(TIFF)
