# Supplementary material for: Breaking Snake Camouflage: Humans Detect Snakes More Accurately than Other Animals under Less Discernible Visual Conditions
Source: PLoS One. 2016 Oct 26;11(10):e0164342. doi: 10.1371/journal.pone.0164342 (PMC5081170; doi:10.1371/journal.pone.0164342)
Supplement: S1 File — (DOC) [file pone.0164342.s001.doc]

These are mean luminance levels and root mean square (RMS) contrast of all images in this study.

| Category | # | Mean Luminance level | RMS Contrast |
| --- | --- | --- | --- |
| snake | 1 | 128.2829 | 0.4618 |
|  | 2 | 125.5804 | 0.7107 |
|  | 3 | 127.4372 | 0.4252 |
|  | 4 | 125.7434 | 0.5038 |
| bird | 1 | 127.9273 | 0.2786 |
|  | 2 | 127.5752 | 0.2240 |
|  | 3 | 127.3498 | 0.4390 |
|  | 4 | 127.4019 | 0.4561 |
| cat | 1 | 106.8055 | 0.4339 |
|  | 2 | 126.0924 | 0.4091 |
|  | 3 | 97.8095 | 0.4422 |
|  | 4 | 104.5103 | 0.6504 |
| fish | 1 | 98.0897 | 0.5983 |
|  | 2 | 98.8798 | 0.3011 |
|  | 3 | 98.9195 | 0.6303 |
|  | 4 | 98.1213 | 0.4097 |
