# Supplementary figures and images for: Breaking Snake Camouflage: Humans Detect Snakes More Accurately than Other Animals under Less Discernible Visual Conditions
Source: PLoS One. 2016 Oct 26;11(10):e0164342. doi: 10.1371/journal.pone.0164342 (PMC5081170; doi:10.1371/journal.pone.0164342)

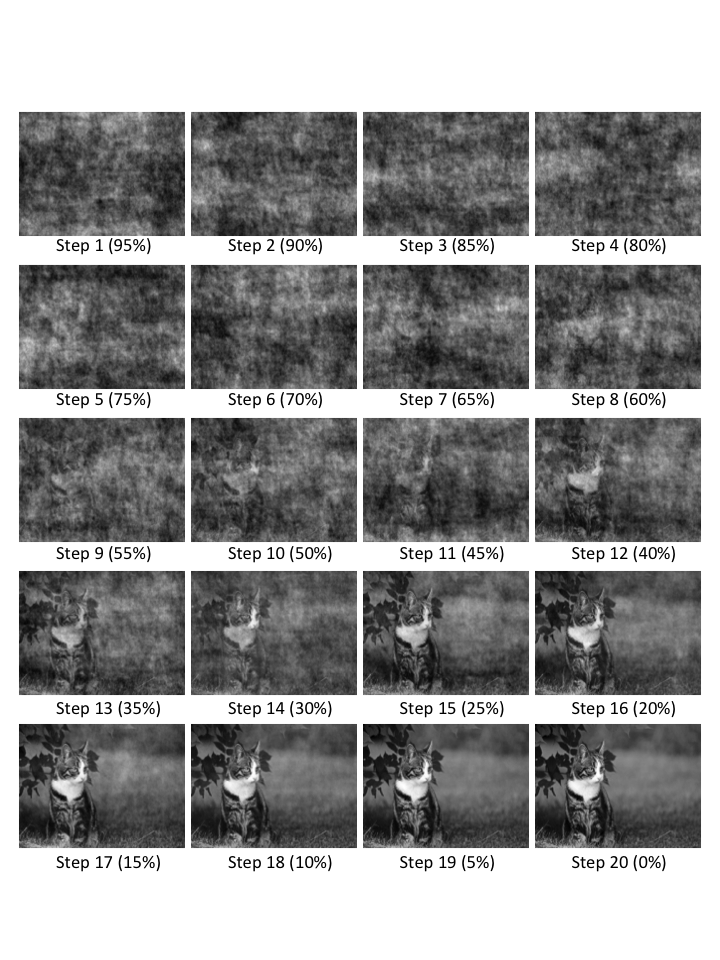

Supplement: S2 File — Examples of a Random Image Structure Evolution (RISE) sequence for cat pictures. Includes a sequence of 20 pictures with interpolation ratio starting from 95% to 0% with steps of 5%. RISE sequence will gradually change from unorganized to well discernible. (TIFF) [file pone.0164342.s002.tiff]
